# The Effect of Slack Resources on Innovation Performance and the Environmental Adaptability of Public Hospitals: The Empirical Evidence From Beijing of China

**DOI:** 10.3389/fpubh.2022.904984

**Published:** 2022-07-01

**Authors:** Wei Lu, Xinrui Song, Changmin Hou, Junli Zhu

**Affiliations:** ^1^School of Public Health, Capital Medical University, Beijing, China; ^2^Research Center for Capital Health Management and Policy, Beijing, China; ^3^Beijing Chest Hospital, Capital Medical University, Beijing, China; ^4^Beijing Jishuitan Hospital, Beijing, China

**Keywords:** public hospital, slack resources, innovation performance, environmental adaptability, mediating effect

## Abstract

**Background:**

The development level of public hospitals has a direct impact on people's health. The reform of the medical industry in China has been gradually underway in recent years, while hospitals face a complex and uncertain environment. This study aims to explore the mechanism of resources slack in buffering environmental uncertainty and promoting innovation in public hospitals.

**Methods:**

Based on previous literature related to environmental adaptability, resources slack, and innovation performance, this study has conducted a literature review and has established a study framework. A questionnaire survey has been conducted among clinicians in representative tertiary public hospitals in Beijing. A total of 318 valid data have been eventually obtained, while regression models have been used to analyze the data.

**Results:**

Innovation performance has played a mediating role in the impact of both resources slack and its three dimensions on environmental adaptability of public hospitals. Among them, there has been a complete mediating effect for time slack, while there has been a partial mediating effect for staff and space dimensions.

**Conclusion:**

This study found that resources slack in public hospitals can improve environmental adaptability by affecting innovation performance. It is necessary for public hospitals to reserve resources slack to ensure that there is sufficient condition for innovation in the face of uncertain changes.

## Introduction

Public hospitals are the main body of the medical service system in China, and they are responsible for meeting the basic needs of the people for medical treatment, while their levels of development have a direct impact on people's health (1). It is what people expect from public hospitals to provide high-quality, efficient, convenient, and affordable medical services through rational and effective use of health resources. However, the healthcare industry is faced with a complex and uncertain environment. Pourmohammadi et al. (2) have divided hospital environmental factors into micro-environmental factors, such as unfair resource allocation and macro-environmental factors, such as political factors. Hospitals need to have the ability to adapt to the new environment.

Affected by the economic system in China, public hospitals have been operating with less competition in the medical market, while the inefficient operation has been criticized for a long time. In recent years, China's economic development has gradually changed, while many industries, including the medical industry, urgently need to transform to high-quality development to form a competitive advantage. Since the new medical reform in 2009, a series of reforms have been carried out to improve the operating mechanism of public hospital (3), including canceling markups on pharmaceuticals and optimizing medical insurance payment models. These reforms have brought the increasing competition to public hospitals and have made them encounter the critical environmental changes.

As the foundation of organizational development, resources not only provide the organization's normal operation needs but also increase its responsiveness in changes. The theory of organization slack has shown that slack resources allow an organization to adapt successfully to internal pressures for adjustment or to external pressures for change in policy (4), while slack in hospitals has a positive effect on quality of medical service (5). Shahzad et al. (6) have proved that slack plays a crucial role in shaping organizational behavior, including innovations, while it is necessary for the medical industry to use slack resources to promote innovation.

A number of policies issued by the National Health Commission have also reflected the importance of innovative behavior in hospitals. “Opinions on Adhering to the People's Health as the Center to Promote the High-Quality Development of Medical Services”(2018) has emphasized that scientific progress and conceptual innovation should be relied on to improve the efficiency of medical services, while “Opinions on Promoting the High-Quality Development of Public Hospitals” (2021) has pointed out that technological innovation, model innovation, and management innovation should be strengthened. “Implementation Plan on Strengthening the Research and Innovation Functions of Medical and Health Institutions,” which was issued by Beijing Municipal Health Commission, has also pointed out that the vitality of scientific research and innovation in medical institutions should be fully stimulated.

The organizational goals of hospitals are different from those of general enterprises, while their performance and achievements are related to people's life and health. It is necessary for us to pay attention to the state and rationality of hospitals' utilization of slack resources. At present, there is still a study gap in the role of resources slack in the medical industry, and the process mechanism of its impact on environmental adaptability and innovation performance is not very clear. And there is no mature evidence in a highly politicized environment like Chinese public hospitals. In view of the above, this study focuses on the impact of slack resources on the innovation performance and environmental adaptability of Chinese public hospitals to fill the above gaps.

## Literature Review

### Definition of Basic Concepts

The concept of environmental adaptability originated from contingency theory, while the traditional concept of environmental adaptation was that the organization scanned customers and competitors to find signals from the environment, filtered information, and then adjusted the organization's activities to cope with changes (7). In order to cope with changing environmental conditions, a more dynamic view of organizational adaptation was needed, and the ability to adapt to the environment should be regarded as a dynamic process of continuous learning (8). This situation also applied to the field of hospital management. In the face of the uncertainty of the hospital environment, an adaptive response strategy could be adopted, that is, the ability to be prepared to allocate resources flexibly (9).

Cybert and March (10) have first defined slack as excess resources clearly, that is, resources exceeding the minimum amount required to complete the task. Slack resources, also known as organizational slack, broadly include redundant inputs, such as employees, production capacity, and non-essential expenditures, as well as unused opportunities, such as gaining additional income and fulfilling social responsibilities (11). Later, Mallidou et al. (12) have introduced the concept of slack into the health field, and literature has divided medical resource slack into time, staff, and space dimensions from the perspective of psychometric assessment, while this study has also adopted this classification.

The traditional concept of innovation referred to the implementation of new products or processes, or new organizational methods in organizations, while literature has emphasized that innovation is the process of transforming new ideas into useful products or services (13). Svensson et al. (14) have shown that the innovation of non-profit organizations was social innovation, which referred to solving social problems or promoting positive social changes through better methods. The public hospitals in China are non-profit organizations and play an important role in all stages of the innovation process, especially in the generation, adoption, and reproduction of medical knowledge (15).

### Relationships Between Variables

#### Resources Slack and Environmental Adaptability

An organization's behavior in the face of environmental changes may be very different from its behavior under normal circumstances. Environmental changes may increase the consumption of internal resources, which would adversely affect the service quality (5). The buffering effect of slack resources protects the organization from the impact of environmental changes, while it can enable the organization to successfully adjust internal pressures and initiate strategies related to the external environment (16). In countries with low levels of sustainable development, enterprises with a large amount of slack resources were better able to cope with pressure (17), while the practical role of slack resources has been controversial in the healthcare field (18). Some believes that slack resources mean too much input or insufficient output, resulting in inefficiency; others focus on the potential to provide hospital managers more opportunities to take actions. Sufficient slack resources can enable hospital administrators to tally with the escalating external condition over time (19). Flexibility is particularly important for the daily operation of a hospital that requires a high degree of coordination, and slack resources can provide more flexibility for the overall operation of the hospital (5). It can be speculated that resources slack and its three dimensions are conducive to the adaptation of hospitals to uncertain environments. Thus, this study proposes the following hypothesis:

*H1*_(*a*/*b*/*c*)_*: Resources (time/staff/space) slack positively affects environmental adaptability*.

#### Resources Slack and Innovation Performance

The existence of resources slack has promoted the development of innovation projects. These projects may bring good benefits to an organization. Cybert and March (10) have proposed that slack resources play an important role in supporting the experimentation of some new strategies and innovative projects, while slack resources provide organizations with the proactive ability to adopt new technologies. In the healthcare industry, innovation refers to the introduction of new medical practices to improve services, such as treatment and diagnosis. Lenz (20) has argued that staff slack, time slack, and space slack not only affect the quality of treatment level and performance indicators of hospitals but also have the potential to support organizational innovation. When healthcare workers face their hospital environment and consider whether they are ready to adopt new practices, slack may help increase acceptance. It can be speculated that resources slack is conducive to innovative behavior.

*H2*_(*a*/*b*/*c*)_*: Resources (time/staff/space) slack positively affects innovation performance*.

#### Innovation Performance and Environmental Adaptability

Environmental uncertainty could be the tendency to create opportunities and benefits (20), while coping with the difficulties caused by environmental dynamics has been also an important goal of innovation. Reducing the negative feelings of uncertainty caused by rapid changes in the market, technology, and knowledge is a powerful driving force to guide organizations to adapt (21), while continuous efforts for uncertain results have been one of the characteristics of innovation (22). The medical and healthcare industry has faced a challenging environment, which forces hospitals to seek new technologies to optimize resources (23). As a knowledge-intensive organization, innovation has been the key element in improving hospital's environmental adaptability and competitive advantage. Schultz et al. (24) have proposed that hospitals need innovation behavior to cope with the pressure of service quality and create technology opportunities. Thus, this study proposes the following hypothesis:

*H3: Innovation performance has a significant positive impact on environmental adaptability*.

### Study Framework

**Figure 1:**
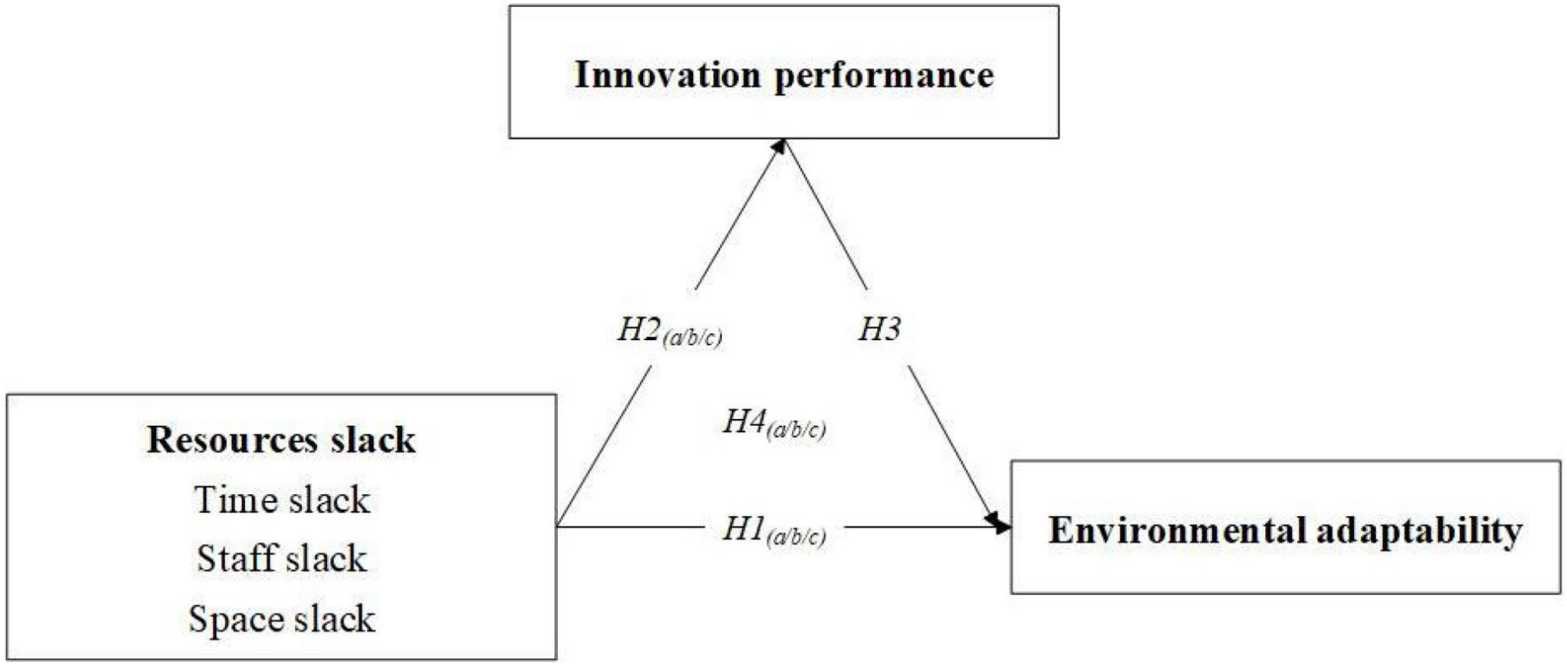
A study framework.

Based on the review of previous literature, this research under the framework of behavioral decision theory, starting from the environment, resources and innovation, proposes hypotheses and conducts empirical verification along the path of problem search, response, and solution. As shown in Figure 1, it can be considered that innovation performance has mediated the process of the impact of public hospital resources slack on environmental adaptability, while the role of resources slack in the three dimensions can also follow this framework. In other words, this study can make the following hypothesis based on the above situation.

*H4*_(*a*/*b*/*c*)_*: Innovation performance plays a mediating effect in the influence of resources* (*time/staff/space*) *slack on environmental adaptability*.

## Methods

### Sample and Data

This study mainly uses questionnaires to collect data. The electronic questionnaire system is used to publish questionnaires online. This research is an anonymous survey and does not contain any identifiable information. Regarding the respondents, clinicians in public hospitals have been selected as the subjects of the questionnaire. Clinicians have a more comprehensive and accurate understanding of the hospital's first-line resources status, and directly use resources to provide medical services, which can ensure the accuracy of information. All the respondents participated voluntarily and signed informed consent. According to the total number of clinicians in each hospital, the sampling proportion and the number of samples in each target hospital have been determined, and a sufficient number of respondents have been randomly selected in each hospital. The questionnaires were issued from March to May 2021, and finally obtained data from clinicians in eleven tertiary public hospitals in Beijing, including six comprehensive hospitals and five specialized hospitals. The invalid questionnaires that have been filled in more than 5 items of the same option were eliminated; 318 valid questionnaires have been finally obtained.

### Instruments

In order to ensure the rationality of the questionnaire, this study has taken a series of measures to improve quality. Firstly, this study has translated the scale currently in English into Chinese, and replaced some expressions with public hospitals according to the research theme. Secondly, a group of 5 experts was formed to discuss the translation and modification of the initial measurement items. Thirdly, before the formal investigation, this study had selected a hospital to conduct a small sample pre-survey, and then several revisions had been made. The content of the final questionnaire included two parts: basic information and scales. The basic information is the characteristics of hospitals and individuals, and the scales in the questionnaire use Likert's 7-level scale to score variables. There are 7 levels of options, ranging from completely disagree/very dissatisfied to completely agree/very satisfied.

#### Measure of Environmental Adaptability

The hospital environment has been becoming more and more uncertain in recent years, while the governance and operation of the Chinese public hospitals involved in this study are led by the government, supervised and compensated through financial subsidies (25). The environmental changes faced are mainly due to the series of medical system reform policies initiated by the government in recent years. Based on the above current situation, this study has designed a question to evaluate the adaptability of hospital clinicians to healthcare reform environment, expressed as “The hospital is fully controllable for the series of changes brought about by the reform of the medical system in the past 5 years.”

#### Measure of Resources Slack

Based on practical significance, this study has measured slack resources that can be adjusted and configured in public hospitals, while the scale is derived from Mallidou et al. (12). The scale has divided resources slack into three dimensions: time slack (4 items), staff slack (2 items), and space slack (3 items). The respondents have answered items, such as ”Do you often have time to do something extra for patients“ and ”Does your department have enough staff to get work done.“ This scale has shown high reliability (Cronbach's α is 0.87), while the Cronbach's α of three subscales is 0.74, 0.90, and 0.89, respectively. And this scale has also shown high validity (KMO value is 0.79), while the KMO values of three subscales are 0.67, 0.50, and 0.73, respectively.

#### Measure of Innovation Performance

The innovation performance scale used in this study is derived from Acosta-Prado et al. (26) on measuring innovation performance of Colombian non-profit hospitals. The scale includes three items, such as ”In the past 5 years, our hospital has provided users with new services,” while the scale has showed high reliability (Cronbach's α is 0.93) and validity (KMO value is 0.75).

#### Control Variables and Selection Basis

This study has selected two types of variables as the control variables. One of the variables is hospital characteristic, including hospital nature, beds, location, and co-workers' numbers, while the other is personal characteristics, including sex, age, job title, working years, and working hours per week.

### Statistical Analysis

Stata 16.0 has been used to analyze the data. Mean and standard deviation have been used on descriptive research for quantitative data, while frequency and percentage have been used on descriptive research for qualitative data. Correlation analysis has been performed to obtain preliminary relationships between variables. To verify the four conditions of the mediation hypothesis, multiple linear regression models have been established to explore the relationship between variables. The relationship of variables in the regression model is as follows:

(1) “*Environmental adaptability”* = β_11_“*Resources slack”*+ β_01_
(2) “*Innovation performance”* = β_12_ “*Resources slack”* + β_02_
(3) “*Environmental adaptability”* = β_13_ “*Innovation performance”* + β_03_
(4) “*Environmental adaptability”* = β_14_ “*Resources slack”* + β_24_ “*Innovation performance”* + β_04_

If the regression coefficients β_11_~β_13_ are significant, and the regression coefficient β_24_ is significant, but β_14_ is not significant (or significant but less than β_12_), it can be considered that innovation performance has a mediating effect on the influence of resources slack on environmental adaptability. If β_14_ is not significant at all, it indicates that innovation performance has played a completely mediating effect, while, if β_14_ is only reduced but still significant, it is only a partial mediating effect.

## Results

### Descriptive Statistics Results

#### Basic Characteristics of Respondents

The descriptive statistics of basic characteristics have been shown in Table 1. Most of the respondents are women (56.92%), who come from general hospitals (50.94%). The average age of them is 34.16 years old, while 16.04% of their job titles are senior. They have worked for an average of 7.46 years and 55.80 h per week in hospitals.

**Table 1:** Descriptive statistics of basic characteristics.

**Hospital variables**	***N* (%)**	**Personal variables**	***N* (%) / M ±SD**
Hospital nature		Sex	
General hospital	162 (50.94)	Female	181 (56.92)
Specialist hospital	156 (49.06)	Male	137 (43.08)
Hospital beds
Below 1000 beds	122 (38.36)	Age	34.16 ± 6.962
1000 beds or more	196 (61.64)		
Hospital location		Job title	
Core functional	211 (66.35)	Senior	51 (16.04)
Function expansion	107 (33.65)	Intermediate	124 (38.99)
Number of co-workers		Junior and below	143 (44.97)
≤ 10	65 (20.44)		
11~20	90 (28.30)	Working years	7.46 ± 6.542
21~50	84 (26.42)		
51~100	43 (13.52)	Working hours per week	55.80 ± 14.333
>100	36 (11.32)		

#### Main Variables Correlations and Comparisons

The scales scores for regression models and correlation analysis of main variables have been shown in Table 2. Checking the correlation coefficient matrix of variables can be considered that there is almost no severe multicollinearity, while environmental adaptability is significantly correlated with resources slack and innovation performance.

**Table 2:** Descriptive statistics and correlation analysis of main variables.

**Variables**	**M ± SD**	Correlations				
		**Environmental adaptability**	**Resources slack**	**Time slack**	**Staff slack**	**Space slack**
Environmental adaptability	4.35 ± 1.465	1				
Resources slack	36.83 ± 10.699	0.328	1			
Time slack	17.86 ± 4.756	0.190	/	1		
Staff slack	7.84 ± 3.422	0.312	/	0.376	1	
Space slack	11.13 ± 4.951	0.311	/	0.449	0.657	1
Innovation performance	14.99 ± 4.168	0.472	0.368	0.295	0.305	0.302

### Regression Analysis Results

#### The Regression Model Based on the Overall Resources Slack Level

The regression model results based on the overall resources slack level is shown in Table 3. Model 0 with innovation performance as the dependent variable has shown a significant impact of resources slack. In addition, five models have been established with environmental adaptability as the dependent variable. Model 1, which only includes control variables, has shown that hospital bed is the only significant factor. And Model 2 has used three dimensions of slack as independent variables simultaneously, of which staff and space dimensions are significant factors. Model 3 has used innovation performance as an independent variable and finds that it is statistically significant (*p* < 0.01). Model 4 uses the total score of resources slack as an independent variable, which is also a significant factor (*p* < 0.01). Model 5 has taken innovation performance and resources slack as independent variables at the same time, while both are significant factors. The regression coefficient of resources slack is smaller than innovation performance, which is also smaller than that of Model 4. It can be considered that innovation performance has a partial mediating effect on the influence of resources slack on environmental adaptability.

**Table 3:** The regression model based on the overall resources slack level.

**Variables**	**Model0^**α**^**	**Model1^**β**^**	**Model2^**β**^**	**Model3^**β**^**	**Model4^**β**^**	**Model5^**β**^**
Resources slack	0.168***				0.0455***	0.0213***
Innovation performance				0.167***		0.144***
Time slack			0.00684			
Staff slack			0.0807***			
Space slack			0.0574**			
Hospital nature	3.116***	0.0557	0.407	−0.297	0.327	−0.122
Hospital beds	2.063**	−0.554*	−0.308	−0.766***	−0.339	−0.636**
Hospital location	−1.023*	−0.224	−0.388*	−0.147	−0.377*	−0.230
Sex	−0.603	0.0519	−0.0794	0.100	−0.0328	0.0541
Age	0.170**	0.0333	0.0319	0.00472	0.0328	0.00838
Working years	−0.102	−0.0396	−0.0289	−0.0154	−0.0280	−0.0132
Job title	0.443	−0.211	−0.117	−0.217	−0.101	−0.165
Working hours per week	0.0271*	−0.00211	0.00234	−0.00435	0.00161	−0.00231
Number of co-workers	0.131	−0.0387	−0.0491	−0.0661	−0.0477	−0.0666
constant	−1.217	4.574***	2.518**	3.332***	2.226*	2.402**
*R^2^*	0.219	0.057	0.174	0.272	0.158	0.290
Adjusted *R^2^*	0.193	0.029	0.141	0.248	0.131	0.264

N = 318, ^α^innovation performance as a dependent variable, ^β^environmental adaptability as a dependent variable, *p <0.1, **p <0.05, ***p <0.01.

#### The Regression Model Based on the Time, Staff, and Space Slack Levels

The regression models based on time, staff, and space dimensions of resources slack have been respectively shown in Table 4. In order to verify the mediation effect, innovation performance has been first used as the dependent variable, and three dimensions have been included as independent variables into Model 0. The three dimensions are all significant (*p* < 0.01). Models 1, 2, and 3 have all used environmental adaptability as the dependent variable. Model 1 has shown that innovation performance significantly affects environmental adaptability. Model 2 has taken time, staff, and space dimensions as independent variables, in turn, while they all significantly affect environmental adaptability (*p* < 0.01). Time slack has the lowest regression coefficient, and staff slack has the highest one. Model 3 takes innovation performance and three types of slack into the models in turn. Among them, innovation performance is a significant factor in each model (*p* < 0.01); staff slack and space slack still significant (*p* < 0.01), but the regression coefficients are significantly lower than Model 2, while time slack is not significant. The above results show the mediating effect of innovation performance, which has partial mediating effect on staff slack and space slack, and complete mediating effect on time slack.

**Table 4:** The regression model based on the three dimensions of the slack level.

**Variables**	**Model0^α^**			**Model1^**β**^**	**Model2^β^**			**Model3^β^**		
	**(1)**	**(2)**	**(3)**		**(1)**	**(2)**	**(3)**	**(1)**	**(2)**	**(3)**
Time slack	0.272***				0.0528***			0.00822		
Staff slack		0.424***				0.137***			0.0751***	
Space slack			0.322***				0.0970***			0.0502***
Innovation performance				0.167***				0.164***	0.146***	0.145***
Hospital nature	2.253***	2.748***	3.389***	−0.297	0.0828	0.260	0.440	−0.287	−0.141	−0.0528
Hospital beds	1.508*	1.620*	2.241**	−0.766***	−0.508	−0.441	−0.261	−0.755***	−0.677**	−0.587**
Hospital location	−0.707	−0.780	−1.002*	−0.147	−0.272	−0.328	−0.388*	−0.156	−0.214	−0.242
Sex	−0.271	−0.673	−0.621	0.100	0.0556	−0.0716	−0.0478	0.100	0.0266	0.0425
Age	0.175**	0.170**	0.165**	0.00472	0.0340	0.0327	0.0314	0.00533	0.00798	0.00742
Working years	−0.115	−0.118*	−0.118*	−0.0154	−0.0339	−0.0310	−0.0314	−0.0149	−0.0137	−0.0143
Job title	0.370	0.353	0.180	−0.217	−0.147	−0.109	−0.168	−0.207	−0.161	−0.194
Working hours/week	0.0186	0.0266	0.0236	−0.00435	−0.00111	0.00215	0.000945	−0.00416	−0.00173	−0.00248
Number of co-workers	0.137	0.0895	0.189	−0.0661	−0.0440	−0.0628	−0.0311	−0.0665	−0.0759	−0.0586
constant	1.122	2.521	2.062	3.332***	3.346***	2.984**	2.952**	3.162***	2.616**	2.653**
*R^2^*	0.141	0.161	0.180	0.272	0.085	0.152	0.154	0.272	0.297	0.294
Adjusted *R^2^*	0.113	0.134	0.153	0.248	0.055	0.125	0.126	0.246	0.271	0.268

N = 318, ^α^innovation performance as a dependent variable, ^β^environmental adaptability as a dependent variable, *p <0.1, **p <0.05, ***p <0.01.

## Discussion

This study has deepened how public hospitals can conduct innovative behaviors to better utilize resources slack to deal with environmental uncertainty and improve adaptability. From the results, the hypotheses have been supported by a series of evidence.

Public hospitals' resources slack has a significant positive correlation with environmental adaptability, while the mechanism can be explored from two aspects. In one aspect, resources slack provides the organizational foundation and has a buffering effect. Laffranchini and Braun (27) have said that resource slack allows hospitals not to think too much about the risk, because there are additional resources to offset the loss caused by failure. In the face of endless medical reform policies in recent years, the hospital can use the resources that are not normally put into use to replace the resources lost in time to maintain the operation of the hospital. In the other aspect, this study has shown that the three dimensions of slack in public hospitals are all useful for responding to uncertain environmental changes, such as medical policy reforms. The impact of staff slack is the most critical, followed by space slack. Azadegan et al. (28) have shown that resources slack provides flexibility in the form of surplus labor or available inventory, enabling enterprises to respond to changes faster and more effectively. Staff and space, which are the foundation of an organization, can be taken as the premise of time, and only when someone has space can he or she put the extra time into use. Kerfoot (29) has also emphasized that excellent hospital staffs in hospitals are not always busy but should be good at thinking and soliciting solutions.

This study has confirmed that innovation performance plays a mediating effect on the effect of resources slack on environmental adaptability. According to the organizational behavior theory, when an enterprise faces a dynamic environment, slack resources can allow organizations to try more innovations to avoid crises and improve response capabilities (30). The actual innovation activities take place in a complex environment, while it is necessary to consider not only the resources slack within the organization that can support innovation behavior but also the excitation of innovation by external factors, such as environmental uncertainty (31). This can also be confirmed in the field of hospital management, such as Salge (32), which has proposed that the innovation behavior of public hospitals includes two modes: one mode is a problem-based mode, which is triggered by negative environmental feedback and awareness of performance deficit to explore solutions, while the other mode is slack based, which is triggered by decision makers seeking the best use of available slack resources (32). Slack resources are not only the main driving force of the slack-based model but also an important prerequisite for public service organizations to implement the problem-based model. George et al. (33) have combined innovation with prospect theory and threat rigidity hypothesis, and have pointed out that hospitals are, often, more willing to seek solutions that are different from conventional behaviors when faced with environmental changes. But the gradual reduction of discretionary resources will cause hospitals to reduce the diversity of solutions. There is no doubt that innovation performance plays a buffering role in public hospitals by transforming resources slack.

The hospital's innovative performance is significantly positively correlated with its adaptability to uncertain environments in this study. Tuominen et al. (34) have provided a strong theoretical foundation for the relationship by integrating a competency-based view of competition and a knowledge-based innovation management theory. In the context of revitalizing China by applying scientific and technological advances, the competitiveness of organizations mainly comes from innovative activities (31). In educational institutions, Shen (35) has pointed out that innovation is driven by internal and external environments, and the ultimate goal is to make educational practices more effective. Innovation can effectively and fully utilize the knowledge and resources and promote the optimization and upgrade of operational capabilities, so as to ensure that the hospitals can face the uncertain policy environment, such as cancellation of the medicine markup and reforming Medicare payments that drastically affect the way of income. In addition, Yamakawa and Ostos (36) have pointed out that uncertain environment can promote the organization to stimulate the motivation of technological innovation. From the above, the two forces of environmental and innovation may be interacting.

Resources slack contributes to the improvement of innovative behavior in this study, which is similar to Wang and Cheng (37)'s findings of China. Discretionary slack resources can help organizations seize opportunities to implement innovative behaviors and ensure continued investment and support at different stages of the innovation process (38). To carry out breakthrough innovations, Troilo et al. (39) have pointed out that the organization must mobilize additional resources to integrate new technologies and develop new capabilities. In recent years, studies have suggested that slack resources may have differential effects on different concepts of innovation, and that different specific forms of slack may also lead to different outcomes (40, 41). Pollock et al. (40) have believed that certain forms of slack resources may play different roles in different organizational behaviors and results, and special attention should be paid to time slack. The result of this study has confirmed that time slack is affected by the environment adaptability entirely through the channel of innovation performance, while innovation performance also plays a partial mediating role in the impact of staff slack and space slack on environmental adaptability. Shen (35) has said that innovation in educational institutions, such as schools, requires sufficient time and personnel, with teachers as the main facilitators and students as the focal point. Kerfoot (29) has suggested that staffs do not have time to think and analyze in most cases due to work pressure, and it is difficult to propose and establish new working models in a busy organizational culture, which inhibits the innovative behavior of hospital administrators and doctors. Using time slack for in-depth thinking also opens up opportunities for the growth of creativity and professional practice. Acosta-Prado et al. (26) have believed that the impact of human resources on the performance of non-profit organizations such as hospitals is positive, and reasonable investment in staff may be more conducive to innovation. And Mallidou et al. (12) have argued that the mechanism of hospital slack resources may be beneficial to managers and professionals to have additional space to develop or implement innovative ideas. These two dimensions may also affect environmental adaptability through other channels, so innovation performance only has a partial mediating effect of these two dimensions.

This study has some contributions. First, unlike most studies based on for-profit corporations, this study looks at typical non-profit organizations—public hospitals. In addition, the respondents in this study are clinicians in public hospitals, and their perception is more intuitive than that of managers. Second, unlike earlier studies, which were mostly based on hospitals in developed countries, such as Germany (32), this study provides evidence at the level of special political environment. China's public hospitals are in a highly politicized environment, and their behaviors are greatly interfered by the government. However, the current performance appraisal mechanism is hard to allow a lot of resources slack to be left. Third, this study has pioneered the selection of hospital innovation performance as a mediating variable to study its role in the process of slack resources affecting environmental adaptation. It has practical significance for government policy formulation and hospital management.

This study also has some limitations, which may be resolved in future studies. First, this study is limited to the study of tertiary public hospitals in Beijing, which has limited representativeness. Selecting hospitals of different levels and locations may be considered for further and more general research in the future. Second, this study is a cross-sectional study, which cannot reflect the process of hospitals responding to changes in the medical reform policy environment, while the method of more advanced models is not adopted. Follow-up investigations may be considered to provide an in-depth analysis of this dynamic process in the future. Third, subjective evaluations are closely related to the individual perception ability, but do not reflect the objective situation of hospital resources and environmental changes. A future study can consider adopting the perspective of hospital managers or superiors to objectively evaluate innovation performance or environmental adaptability, and the objective level of resources slack through the actual resources owning the situation of the hospital can also be estimated.

## Conclusion

This study has established a study framework under the literature review, and this study has conducted a cross-sectional survey with Chinese public hospitals as the research object. Through the construction of regression models, this study has confirmed the impact of resources slack on innovation performance and environmental adaptation, and has found that innovation performance played a mediating role in the impact of resources slack on environmental adaptation. This study fills a gap where resources slack in the healthcare industry functions in a highly politicized environment, emphasizing the importance of hospitals deploying appropriate slack resources and encouraging innovative behavior. And this study has certain reference significance for public hospital managers and clinicians how to better allocate health resources. However, this study also has limitations in terms of hospital types and methods. In the future, other types of hospitals, more objective measurement methods, or cohort studies may be considered for a further study.

## Data Availability Statement

The raw data supporting the conclusions of this article will be made available by the authors, without undue reservation.

## Ethics Statement

The protocol of this study was approved by the Medical Ethics Committee of Capital Medical University (No: Z2020SY123). All the respondents were voluntary, and written informed consent was obtained. All data collection is anonymous.

## Author Contributions

WL and JZ contributed to the conception and design of the study. XS, CH, and JZ organized the data collection. WL and XS performed the statistical analysis. WL wrote the content of the manuscript. All authors contributed to the manuscript revision, read, and approved the submitted version.

## Funding

This research was supported by the National Natural Science Foundation of China (Grant Nos. 71573182 and 71974133).

## Conflict of Interest

The authors declare that the research was conducted in the absence of any commercial or financial relationships that could be construed as a potential conflict of interest.

## Publisher's Note

All claims expressed in this article are solely those of the authors and do not necessarily represent those of their affiliated organizations, or those of the publisher, the editors and the reviewers. Any product that may be evaluated in this article, or claim that may be made by its manufacturer, is not guaranteed or endorsed by the publisher.

## References

[1] Zhang C Liu Y. The salary of physicians in Chinese public tertiary hospitals: a national cross-sectional and follow-up study. BMC Health Serv Res. (2018) 18:661. 10.1186/s12913-018-3461-7 30143042 PMC6109324

[2] Pourmohammadi K Bastani P Shojaei P Hatam N Salehi A. A comprehensive environmental scanning and strategic analysis of Iranian public hospitals: a prospective approach. BMC Res Notes. (2020) 13:179. 10.1186/s13104-020-05002-8 32216824 PMC7098121

[3] Wei Q Pan X. Analysis of the unified management mode of medical insurance and prices in large public hospitals. Health Econ Res. (2012) 11:50–1.

[4] Bourgeois LJ. On the measurement of organizational slack. Acad Manage Rev. (1981) 6:29–39. 10.5465/amr.1981.4287985

[5] Youn KI Wan TT. Effects of environmental threats on the quality of care in acute care hospitals. J Med Syst. (2001) 25:319–31. 10.1023/A:1010637324420 11508905

[6] Shahzad AM. Mousa FT, Sharfman MP. The implications of slack heterogeneity for the slack-resources and corporate social performance relationship. J Bus Res. (2016) 69:5964–71. 10.1016/j.jbusres.2016.05.010

[7] Weick KE. The Social Psychology of Organizing. New York, NY: McGraw-Hill, Inc (1979).

[8] Staber U. Sydow J. Organizational adaptive capacity: a structuration perspective. J Manag Inq. (2002) 11:408–95. 10.1177/1056492602238848

[9] Tolf S Nyström ME Tishelman C Brommels M Hansson J. Agile, a guiding principle for health care improvement? Int J Health Care Qual Assur. (2015) 28:468–93. 10.1108/IJHCQA-04-2014-0044 26020429

[10] Cybert RM March JG. A Behavioral Theory of the Firm. Englewood Cliffs: Prentice Hall (1963).

[11] Zhou J Xue Q. An analysis of the research frontier of ambidextrous organization construction. Foreign Econ Manag. (2009) 31:50–7.

[12] Mallidou AA Cummings GG Ginsburg LR Chuang YT Kang S Norton PG. Staff, space, and time as dimensions of organizational slack: a psychometric assessment. Health Care Manage Rev. (2011) 36:252–64. 10.1097/HMR.0b013e318208ccf8 21646884

[13] Amabile TM. A model of creativity and innovation in organization. Res Organ Behav. (1988) 10:123–67.

[14] Svensson G Mahoney T Hambrick M. What does innovation mean to nonprofit practitioners? International insights from development and peacebuilding nonprofits. Nonprofit Voluntary Sec Q. (2019) 49:380–98. 10.1177/0899764019872009

[15] Thune T Mina A. Hospitals as innovators in the health-care system: a literature review and research agenda. Res Policy. (2016) 45:1545–57. 10.1016/j.respol.2016.03.010

[16] Oviatt BM. Agency and transaction cost perspectives on the manager-shareholder relationship: incentives for congruent interests. Acad Manage Rev. (1988) 13:214–25. 10.5465/amr.1988.4306868

[17] Xiao C Wang Q Van Donk DP Taco VDV. When are stakeholder pressures effective? an extension of slack resources theory. Int J Prod Econ. (2018) 199:138–49. 10.1016/j.ijpe.2018.03.002

[18] Zinn J Flood AB. Commentary: Slack resources in health care organizations - fat to be trimmed or muscle to be exercised? Health Serv Res. (2009) 44:812–20. 10.1111/j.1475-6773.2009.00970.x 19674426 PMC2699909

[19] Towers TJ Clark J. Pressure and performance: buffering capacity and the cyclical impact of accreditation inspections on risk-adjusted mortality. J Healthcare Manag. (2014) 59:323–35. 10.1097/00115514-201409000-00005 25647951

[20] Lenz R. Environmental, strategy, organization structure and performance: patterns in one industry. Strateg Manag J. (1980) 1:209–26. 10.1002/smj.425001030315923929

[21] Beckman CM. Haunschild PR, Phillips DJ. Friends or strangers? firm-specific uncertainty, market uncertainty, and network partner selection. Organ Sci. (2004) 15:259–75. 10.1287/orsc.1040.0065

[22] Yang CW Yan YH Fang SC Inamdar SN Lin HC. The association of hospital governance with innovation in Taiwan. Int J Health Plan Manag. (2018) 33:246–54. 10.1002/hpm.2441 28643470

[23] Wiggins D. Financial management, the Web way. Health Manag Technol. (2007) 28:22–4. 17256454

[24] Schultz C Zippel-Schultz B Salomo S. Hospital innovation portfolios: key determinants of size and innovativeness. Health Care Manage Rev. (2012) 37:132–43. 10.1097/HMR.0b013e31822aa41e 21799435

[25] Xue L. Analysis of the external environment of public hospitals' governance innovation. Med Soc. (2014) 27:54–6.

[26] Acosta-Prado JC López-Montoya OH Sanchís-Pedregosa C Zárate-Torres RA. Human resource management and innovative performance in non-profit hospitals: the mediating effect of organizational culture. Front Psychol. (2020) 11:1422. 10.3389/fpsyg.2020.01422 32636791 PMC7318991

[27] Laffranchini G Braun M. Slack in family firms: evidence from Italy (2006-2010). J Fam Bus Manag. (2014) 4:171–93. 10.1108/JFBM-04-2013-0011

[28] Azadegan A Patel PC Parida V. Operational slack and venture survival. Prod Oper Manag. (2013) 22:1–18. 10.1111/j.1937-5956.2012.01361.x

[29] Kerfoot K. Beyond busyness: creating slack in the organization. Medsurg Nurs. (2007) 16:61–3. 17441635

[30] Argilés-Bosch JM Garcia-Blandón J Ravenda D Martinez-Blasco M. An empirical analysis of the curvilinear relationship between slack and firm performance. J Manag Control. (2018) 29:361–97. 10.1007/s00187-018-0270-4

[31] Na R Cheng W. Research on the impact of government subsidies on enterprise innovation: the interactive moderating effect of precipitation redundancy and environmental uncertainty. J Sichuan Univ Technol. (2009) 34:20–34.

[32] Salge TO. A behavioral model of innovative search: evidence from public hospital services. J Public Adm Res Theory. (2011) 30:181–210. 10.1093/jopart/muq017

[33] George E Chattopadhyay P Sitkin SB Barden J. Cognitive underpinnings of institutional persistence and change: a framing perspective. Acad Manag Rev. (2007) 31:347–65. 10.5465/amr.2006.20208685

[34] Tuominen M Rajala A Moller K Anttila M. Assessing innovativeness through organisational adaptability: a contingency approach. Int J Technol Manag. (2003) 25:643–58. 10.1504/IJTM.2003.003129

[35] Shen Y. The effect of changes and innovation on educational improvement. Int Educ Stud. (2008) 1:73–7. 10.5539/ies.v1n3p73

[36] Yamakawa TP Ostos MJ. The influence of the environment on organizational innovation in service companies in Peru. Revista Brasileira de Gestao de Negocios. (2013) 15:582–600. 10.7819/rbgn.v15i49.1586

[37] Wang Y Cheng X. Environmental uncertainty, precipitated redundant resources and enterprise innovation–based on empirical evidence of china's manufacturing listed companies. Stud Sci. (2014) 32:1242–50.

[38] Greve HR. A behavioral theory of R&D expenditures and innovations: Evidence from shipbuilding. Acad Manag J. (2003) 46:685–702. 10.2307/30040661

[39] Troilo G Luca LMD Atuahene-Gima K. More innovation with less? a strategic contingency view of slack resources, information search, and radical innovation. J Prod Innov Manag. (2013) 31:259–77. 10.1111/jpim

[40] Pollock T Porac JF Mishina Y. Are more resources always better for growth? resource stickiness in market and product expansion. Soc Sci Electron Pub. (2010) 25:1179–97. 10.1002/smj.424

[41] Voss GB Sirdeshmukh D Voss ZG. The effects of slack resources and environmental threat on product exploration and exploitation. Acad Manag J. (2008) 51:147–64. 10.5465/amj.2008.30767373

